# Roles of *ZnT86D* in Neurodevelopment and Pathogenesis of Alzheimer Disease in a *Drosophila melanogaster* Model

**DOI:** 10.3390/ijms231911832

**Published:** 2022-10-05

**Authors:** Banseok Lee, Byoungyun Choi, Youngjae Park, Seokhui Jang, Chunyu Yuan, Chaejin Lim, Jang Ho Lee, Gyun Jee Song, Kyoung Sang Cho

**Affiliations:** 1 Department of Biological Sciences, Konkuk University, Seoul 05029, Korea; 2 Korea Hemp Institute, Konkuk University, Seoul 06029, Korea; 3 Department of Medical Science, College of Medicine, Catholic Kwandong University, Gangneung 25601, Korea; 4 Translational Brain Research Center, International St. Mary’s Hospital, Catholic Kwandong University, Incheon 22711, Korea

**Keywords:** Alzheimer disease, neuronal development, *Drosophila melanogaster*, *ZnT7*, *ZnT86D*

## Abstract

Zinc is a fundamental trace element essential for numerous biological processes, and zinc homeostasis is regulated by the Zrt-/Irt-like protein (ZIP) and zinc transporter (ZnT) families. ZnT7 is mainly localized in the Golgi apparatus and endoplasmic reticulum (ER) and transports zinc into these organelles. Although previous studies have reported the role of zinc in animal physiology, little is known about the importance of zinc in the Golgi apparatus and ER in animal development and neurodegenerative diseases. In this study, we demonstrated that *ZnT86D*, a *Drosophila* ortholog of *ZnT7*, plays a pivotal role in the neurodevelopment and pathogenesis of Alzheimer disease (AD). When *ZnT86D* was silenced in neurons, the embryo-to-adult survival rate, locomotor activity, and lifespan were dramatically reduced. The toxic phenotypes were accompanied by abnormal neurogenesis and neuronal cell death. Furthermore, knockdown of *ZnT86D* in the neurons of a *Drosophila* AD model increased apoptosis and exacerbated neurodegeneration without significant changes in the deposition of amyloid beta plaques and susceptibility to oxidative stress. Taken together, our results suggest that an appropriate distribution of zinc in the Golgi apparatus and ER is important for neuronal development and neuroprotection and that ZnT7 is a potential protective factor against AD.

## 1. Introduction

Zinc is a fundamental trace element essential for the activity of over 300 enzymes and 1000 transcription factors [1]. It is the most abundant heavy metal in the brain and plays an essential role in normal brain functions [2]. Approximately 90% of zinc in the brain exists in a protein-bound state and confers catalytic activity and structural stability [3]. A significant amount of the chelatable zinc is present in the presynaptic vesicles of glutamatergic neurons [2]. They are secreted into synapses and modulate neurotransmission by interacting with a number of zinc-permeable channels [2]. Zinc is also crucial for normal brain development [4]. When zinc is depleted in the early postnatal period, the developmental apoptosis of neurons necessary for proper formation of the central nervous system decreases remarkably in rats [5,6]. In addition, maternal zinc deficiency during pregnancy reduces nestin levels in offspring, which could cause abnormal neurogenesis in mice [7].

It is also well known that heavy metals, such as zinc and copper, are involved in the pathology of Alzheimer disease (AD) [8]. AD is a chronic neurodegenerative disorder that accompanies dementia and progressively worsens over decades [9,10]. Brain lesions of patients with AD are characterized by amyloid beta (Aβ) plaques and neurofibrillary tangles. The exact cause of AD remains unknown, and there is no definitive cure. In particular, zinc is colocalized with Aβ plaques in the brain tissue of patients with AD [11], and zinc binds to Aβ peptide and promotes its aggregation in vitro [12,13]. In addition, increased zinc levels are noted in the cortex and serum of patients with AD compared to those in age-matched controls [14,15]. Clioquinol, a drug that chelates zinc and copper, and PBT-2, a second-generation chelator, have shown promising results in clinical trials [2].

Zinc homeostasis is regulated by two major zinc transporter families: the Zrt-/Irt-like protein (ZIP) family and the zinc transporter (ZnT) family [16]. ZIPs transport zinc into the cytosol by importing it from the extracellular space or exporting it into the cytoplasm from intracellular organelles, thereby increasing the intracellular zinc concentration [16]. By contrast, ZnTs, encoded by the solute carrier family 30 (*SLC30A*) genes, generally decrease the zinc level in the cytoplasm. When the intracellular zinc concentration is elevated, zinc is released into the extracellular matrix or into intracellular compartments through ZnTs [17]. Ten ZnT family members have been identified thus far [17]. ZnT1 is localized to the plasma membrane and releases zinc into the extracellular space [17]. By contrast, ZnT2–8 are embedded in the membrane of intracellular compartments and transport zinc into these compartments [17]. In *Drosophila*, seven ZnT homologs have been identified based on phylogenetic alignments: ZnT49B, ZnT41F, ZnT35C, ZnT33D, ZnT86D, ZnT77C, and ZnT63C [18]. ZnT63C, a homolog of ZnT1, is primarily embedded in the basolateral membrane of enterocytes and transports zinc into the extracellular space, thereby absorbing dietary zinc [19]. ZnT35C, a homolog of ZnT2–4 and ZnT8, is localized on the apical side of the Malpighian tubules [20] and plays a role in the excretion of zinc into the lumen of the tubules [21]. It is also required for zinc storage granule biogenesis in Malpighian tubules [22].

ZnT7 is mainly localized in the Golgi apparatus, and a recent study suggested that ZnT7 is also present in the endoplasmic reticulum (ER), where it transports zinc into organelles [23,24]. Several studies have investigated the physiological role of ZnT7 in *ZnT7*-knockout (KO) mice. For example, *ZnT7*-KO mice were zinc deficient, had low body fat accumulation [25], and manifested impaired glucose tolerance involved with insulin resistance [26]. In addition, ZnT7 was colocalized with Aβ plaques, and there was a significant increase in *ZnT7* expression in the hippocampus and cortex of APP/PS1 transgenic mouse mice [27]. Furthermore, ZnT7 colocalized with Aβ deposits in the brains of patients with AD [28]. 

ZnT86D, a *Drosophila* ortholog of ZnT7, is located in the Golgi apparatus [29] as in mammals, and a few studies have been conducted to investigate the role of ZnT86D in zinc homeostasis. For instance, *ZnT86D* overexpression in neurons resulted in complete developmental lethality [30]. In addition, when *ZnT86D* was overexpressed in the eyes, the eclosed flies displayed depigmentation in the eyes and had flatter eyes than control flies [29]. As such, ZnT86D is thought to play a crucial role in the development of various tissues; therefore, an in-depth study of the role of ZnT86D during development should be conducted.

ZnT86D regulates zinc homeostasis by altering the intracellular zinc distribution. Considering that zinc and AD are closely related, it is highly probable that ZnT86D is involved in AD pathogenesis. For example, the genetic inhibition of *dZIP1*, an ortholog of ZIP1 located in the cell membrane that transports zinc to the cytoplasm, ameliorated the pathology of a *Drosophila* AD model [31]. The expression level of *dZIP1* was altered in the brains of *Aβ42*-overexpressing flies, and when it was silenced in the neurons, the amount of Aβ oligomers and fibril deposits was reduced, ameliorating neurodegeneration and improving cognitive function [31]. As zinc promotes the aggregation of Aβ peptides [12,13], these results were attributed to decreased zinc levels in the brain by downregulating *dZIP1*. Thus, in light of the results of the above study, zinc homeostasis seems to be deeply involved in the pathogenesis of AD. In particular, the zinc distribution inside neurons and the zinc content in the brain are likely to play an important role in this process.

In this study, we examined the role of *ZnT86D* in neuronal development in *Drosophila* as well as in neuroprotection against Aβ toxicity. When *ZnT86D* was silenced in the neurons using *ZnT86D* RNAi, body size, survival rate, and locomotor activity were reduced, and the RNAi flies manifested abnormal neurogenesis and increased cell death. In addition, the downregulation of *ZnT86D* in developing wings interrupted normal wing development. Furthermore, silencing *ZnT86D* in the neurons of a *Drosophila* AD model increased cell death and exacerbated neurodegeneration. These results indicate that zinc distribution between the cytoplasm and Golgi apparatus is crucial for the development of neurons and wings, as well as the pathogenesis of AD.

## 2. Results

### 2.1. Silencing *ZnT86D* in Neurons or Wings Exerted Toxic Effects on the Development of Each Tissue

**Figure 1:**
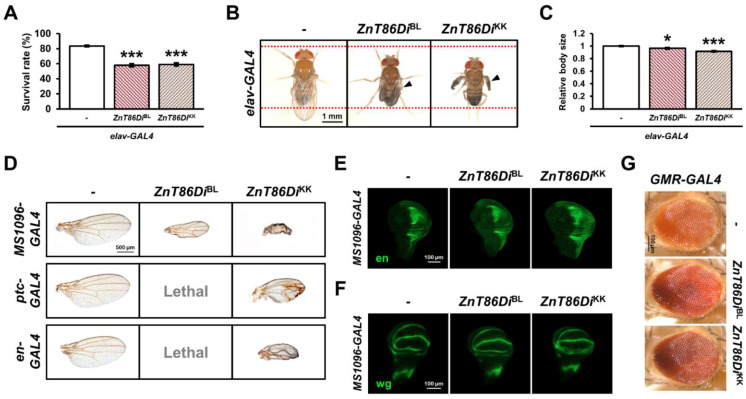
Silencing *ZnT86D* in neurons exerted toxic effects on development of neurons and wings. (**A**) Embryo to adult survival rates of control (*elav-GAL4/+*) and neuron-specific *ZnT86D* RNAi (*elav > ZnT86Di*^BL^ and *elav > ZnT86Di*^KK^) flies (Student’s *t*-test, *n* = 750, *** *p* < 0.001). (**B**) Overall appearance of control and neuron-specific *ZnT86D* RNAi flies. Arrowheads indicate defective wings. (**C**) Relative body size of control and neuron-specific *ZnT86D* RNAi flies (Student’s *t*-test, *n* ≥ 16, * *p* < 0.05, *** *p* < 0.001). (**D**) Wing phenotypes of wing-specific *ZnT86D* RNAi (*MS1096 > ZnT86Di*^BL^ and *MS1096 > ZnT86Di*^KK^; *ptc > ZnT86Di*^BL^ and *ptc > ZnT86Di*^KK^; *en > ZnT86Di*^BL^ and *en > ZnT86Di*^KK^) and respective control (*MS1096-GAL4/+*; *ptc-GAL4/+*; *en-GAL4/+*) flies. (**E**) Representative confocal images showing the engrailed proteins (en) of control (*MS1096-GAL4/+*) and wing-specific *ZnT86D* RNAi (*MS1096 > ZnT86Di*^BL^ and *MS1096 > ZnT86Di*^KK^) flies. (**F**) Representative confocal images showing the wingless proteins (wg) of control (*MS1096-GAL4/+*) and wing-specific *ZnT86D* RNAi (*MS1096 > ZnT86Di*^BL^ and *MS1096 > ZnT86Di*^KK^) flies. (**G**) Eye phenotypes of control (*GMR-GAL4/+*) and eye-specific *ZnT86D* RNAi (*GMR > ZnT86Di*^BL^ and *GMR > ZnT86Di*^KK^) flies. Data are expressed as the mean ± standard error of the mean (SEM).

To investigate the role of *ZnT86D* during *Drosophila* development, we silenced it in the neurons, developing wings and eyes, using several GAL4 drivers. When this gene was silenced in the neurons using *elav-GAL4*, the proportion of individuals emerging from embryos to adults decreased significantly (Figure 1A). In addition, the survived *ZnT86D* RNAi flies had wing expansion defects and reduced body size (Figure 1B,C). 

Furthermore, to examine the role of *ZnT86D* in the wings during development, *ZnT86D* RNAis were expressed under the control of *MS1096-GAL4*, *ptc-GAL4*, and *en-GAL4*. *MS1096-GAL4* drives gene expression in the dorsal wing pouch of the wing imaginal disc during development, *ptc-GAL4* in the anterior/posterior boundary, and *en-GAL4* in the posterior compartment [32,33,34]. When *ZnT86D* was downregulated using the three drivers, crumpled and shrunken wings were observed (Figure 1D). In particular, the *ZnT86Di*^BL^ line was lethal when crossed with *ptc-GAL4* or *en-GAL4* lines (Figure 1D). 

As zinc is a fundamental trace metal essential for the activity of over 1,000 transcription factors [1], changes in intracellular zinc concentration due to *ZnT86D* knockdown may affect the expression of genes that play a pivotal role in wing development, such as engrailed (*en*) and wingless (*wg*). Therefore, we investigated whether changes in the expression patterns of en or wg could be the cause of these results but found no significant changes (Figure 1E,F). This suggests that defects in wing development are not the result of the abnormal expression of genes that are crucial for wing compartmentation. Interestingly, no change in eye shape or size was observed when *ZnT86D* was silenced using *GMR-GAL4* (Figure 1G). Taken together, these data show that *ZnT86D* is essential for the development of neurons and wings, but not eyes.

### 2.2. Neuron-Specific Knockdown of *ZnT86D* Reduced Locomotor Activity and Lifespan, Accompanied by Abnormal Neurogenesis

**Figure 2:**
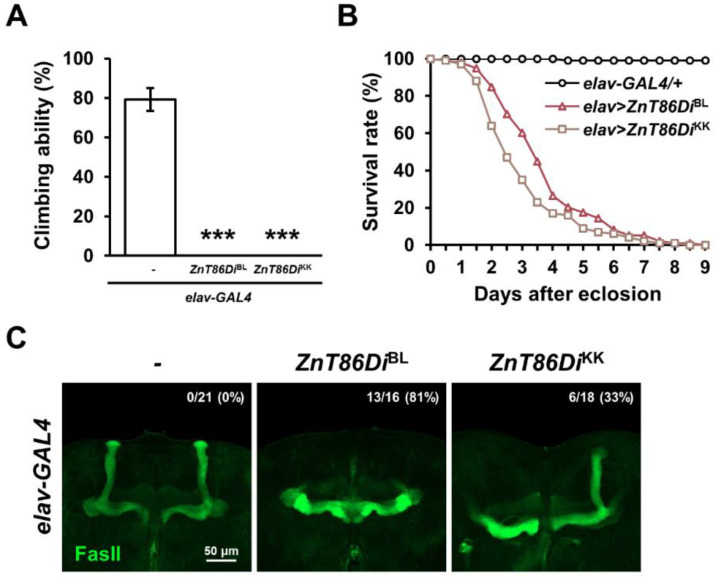
Neuron-specific knockdown of *ZnT86D* reduced locomotor activity and lifespan, accompanied by abnormal neurogenesis. (**A**) Locomotor activities of 2-day-old neuron-specific *ZnT86D* RNAi (*elav > ZnT86Di*^BL^ and *elav > ZnT86Di*^KK^) flies and age-matched control (*elav-GAL4/+*) flies (Student’s *t*-test, *n* = 40, *** *p* < 0.001). Data are expressed as the mean ± SEM. (**B**) Survival curve of control (*elav-GAL4/+*) and neuron-specific *ZnT86D* RNAi (*elav > ZnT86Di*^BL^ and *elav > ZnT86Di*^KK^) flies (Kaplan–Meier estimator and log-rank test, *n* ≥ 98, *elav-GAL4/+* vs. *elav > ZnT86Di*^BL^: *p* = 0; *elav-GAL4/+* vs. *elav > ZnT86Di*^KK^: *p* = 0). (**C**) Representative confocal images showing the mushroom bodies of control (*elav-GAL4/+*) and neuron-specific *ZnT86D* RNAi (*elav > ZnT86Di*^BL^ and *elav > ZnT86Di*^KK^) flies. The numbers at the top refer to the proportion of brains with missing α-lobes to the total number of examined brains in the group.

To further characterize the role of *ZnT86D* in neurons, the locomotor activity of 2-day-old flies was measured using a climbing assay. Surprisingly, the climbing ability of the neuron-specific *ZnT86D* RNAi flies was drastically reduced (Figure 2A). Interestingly, the RNAi flies manifested movements similar to tremors, which can be defined as rhythmic and involuntary movements of any body part [35]. In addition, the flies died within 9 days after eclosion (Figure 2B).

Neurodevelopmental disorders often involve impaired motor function and an increased risk of premature death [36,37]. Therefore, we examined whether the defects caused by the downregulation of *ZnT86D* were associated with neurogenesis by immunostaining mushroom bodies, a center for learning and memory in *Drosophila* brain. Surprisingly, *ZnT86D* RNAi flies had missing α-lobes, implying that neuronal development did not occur normally (Figure 2C). Taken together, these data strongly suggest that *ZnT86D* is essential for neurodevelopment.

### 2.3. Silencing *ZnT86D* in Neurons Increased Cell Death, Which Was Not Associated with ER Stress or Hyperglycemia 

**Figure 3:**
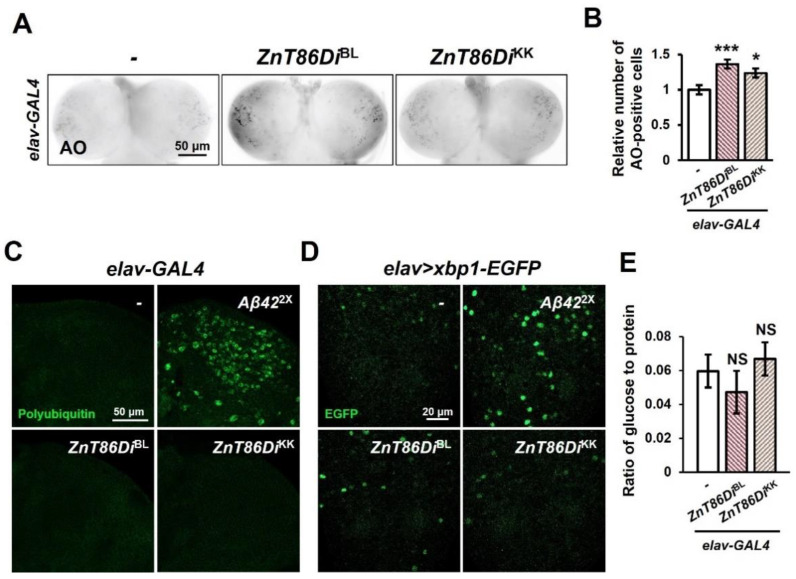
Silencing *ZnT86D* in neurons increased apoptosis. (**A**) Representative fluorescence images showing acridine orange (AO)-positive cells of the larval brains. (**B**) Relative number of AO-positive cells of control (*elav-GAL4/+*) and neuron-specific *ZnT86D* RNAi (*elav > ZnT86Di*^BL^ and *elav > ZnT86Di*^KK^) flies (Student’s *t*-test, *n* ≥ 9, * *p* < 0.05, *** *p* < 0.001). Data are expressed as the mean ± SEM. (**C**) Representative confocal images showing the polyubiquitinated protein aggregates of control and neuron-specific *ZnT86D* RNAi flies stained with FK2 antibody. *Aβ42*-overexpressing flies were used as a positive control. (**D**) Representative confocal images showing the spliced xbp1-EGFP of control and neuron-specific *ZnT86D* RNAi flies. *Aβ42*-overexpressing flies were used as a positive control. (**E**) Circulating glucose levels in the hemolymph of larvae of control and neuron-specific *ZnT86D* RNAi flies (Student’s *t*-test, *N* = 5, NS, not significant). Data are expressed as the mean ± SEM.

To investigate whether the defects caused by the downregulation of *ZnT86D* were associated with increased cell death, we performed acridine orange (AO) staining in the larval brains. As shown in Figure 3A,B, the number of AO-positive cells in the larval brains was increased in the *ZnT86D*-downregulated groups compared to that in the control group, suggesting that the proper level of *ZnT86D* is critical for brain cell survival.

A recent study suggested that ZnT7 is also present in the ER [24], carrying zinc into the organelle, and zinc has been implicated in ER stress and unfolded protein response (UPR) [38]. ER stress occurs when misfolded proteins accumulate and aggregate in the ER, and if these aggregates cannot be properly cleared by UPR, it leads to apoptosis [39]. To test the hypothesis that increased cell death occurred owing to increased protein aggregation, we used an FK2 antibody to verify whether polyubiquitinated protein aggregates were present in the brains of adult flies, but no sign of abnormal protein accumulation was noted (Figure 3C). To confirm that the increased cell death was caused by ER stress, we used xbp1-EGFP, a marker of UPR, in which EGFP is expressed in frame only upon the occurrence of ER stress [40], and there was no significant change in the GFP signal between the *ZnT86D*-downregulated groups and the control group (Figure 3D). Flies overexpressing *Aβ42* in the neurons were used as positive controls in both experiments (Figure 3C,D). 

One of other possible causes of increased brain cell death in neuron-specific *ZnT86D* knockdown flies is increase in blood glucose level. Previous studies have reported metabolic abnormalities in *ZnT7*-KO mice such as glucose intolerance and insulin resistance [26,41,42]. As glucose intolerance is accompanied by hyperglycemia, which increases the risk of diabetes and cardiovascular disease, as well as apoptosis [43], we measured the amount of glucose in the larva hemolymph to determine whether the increased brain cell death is due to increased blood glucose. However, unlike in mice, there was no significant change in the blood glucose levels of *ZnT86D* RNAi larvae compared to control (Figure 3E).

Taken together, neuron-specific knockdown of *ZnT86D* increased cell death in the developing brain, which was not associated with ER stress or high blood glucose level.

### 2.4. Downregulation of *ZnT86D* Exacerbated Aβ Toxicity in a *Drosophila* AD Model

**Figure 4:**
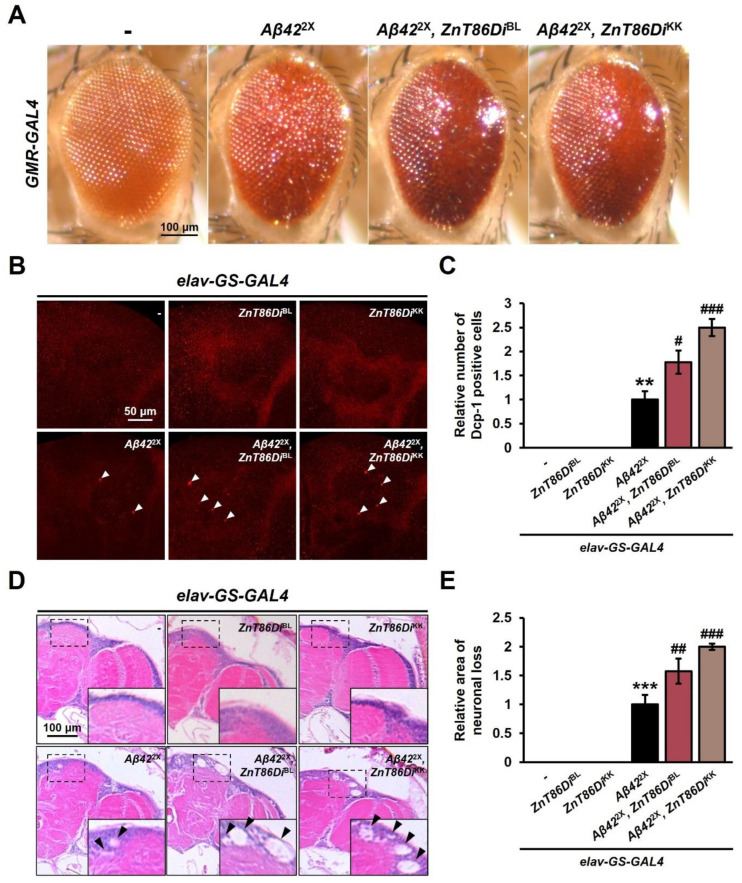
Silencing *ZnT86D* exacerbated Aβ toxicity in a *Drosophila* Alzheimer disease (AD) model. (**A**) Representative images of *Drosophila* eyes showing the effects of *ZnT86D* silencing on the Aβ42-induced rough eye phenotype. (**B**) Representative confocal images showing apoptotic cells detected with anti-active DCP-1 antibody of 20-day-old flies with overexpression of *Aβ42* and reduced expression of *ZnT86D* in neurons (*elav-GS > Aβ42*^2X^, *ZnT86Di*^BL^ and *elav-GS > Aβ42*^2X^, *ZnT86Di*^KK^) and age-matched control flies (*elav-GS > Aβ42*^2X^). Arrowheads indicate active DCP-1-positive cells. To drive the transgene expression, adult flies were raised on standard meal containing 200 μM of RU486. (**C**) Relative number of active DCP-1-positive cells of control group and experimental groups (Tukey–Kramer test, *n* ≥ 7, ** *p* < 0.01, # *p* < 0.05, ### *p* < 0.001). (**D**) Representative images showing neuronal loss in 30-day-old flies with overexpression of *Aβ42* and reduced expression of *ZnT86D* in neurons and age-matched control flies. Arrows indicate vacuoles, which is the area of neuronal loss (**E**) Relative area of neuronal loss of control group and experimental groups (Tukey–Kramer test, *n* = 10, *** *p* < 0.001, ## *p* < 0.01, ### *p* < 0.001). Data are expressed as the mean ± SEM.

It is known that one of the pathological traits of AD is neuronal apoptosis, implicated in neurodegeneration [44,45]. Neuronal apoptosis is induced by the toxic aggregation of Aβ, which eventually leads to neurodegeneration in AD [46,47]. As neuron-specific silencing of *ZnT86D* increased cell death, we investigated the role that *ZnT86D* may play in AD pathogenesis. First, to investigate whether there is a genetic interaction between *ZnT86D* and *Aβ42*, we simultaneously overexpressed *Aβ42* and downregulated *ZnT86D* in the eyes. Interestingly, the Aβ42-induced rough eye phenotype was exacerbated by silencing *ZnT86D* (Figure 4A), but single expression of *ZnT86D* RNAi did not alter the eye phenotype (Figure 1G), suggesting that *ZnT86D* deficiency enhanced Aβ42 cytotoxicity.

Additionally, we investigated the role that *ZnT86D* might have in Aβ-induced apoptosis and neurodegeneration. We used the GeneSwitch (GS) system, which allows temporally and spatially restricted expression of the desired genes [48], thereby excluding the effects of *ZnT86D* RNAi during development. We overexpressed *Aβ42* and downregulated *ZnT86D* simultaneously for 20 days after eclosion in the neurons of adult flies by using *elav-GS-GAL4* and detected cleaved death caspase 1 (active DCP-1) by immunohistochemistry. Unlike when *ZnT86D* was silenced during development, no prominent apoptosis was observed in this model system. However, we found that cell death induced by Aβ42 was exacerbated by downregulating *ZnT86D* (Figure 4B,C). In addition, the degree of neurodegeneration was investigated using histological analysis, and the area of neuronal loss was increased by silencing *ZnT86D* in the brain of 30-day-old flies (Figure 4D,E). Taken together, these data demonstrate that neuron-specific *ZnT86D* downregulation exacerbates Aβ-induced cell death and neurodegeneration.

### 2.5. Knockdown of *ZnT86D* Did Not Increase the Deposition of Aβ Plaques

**Figure 5:**
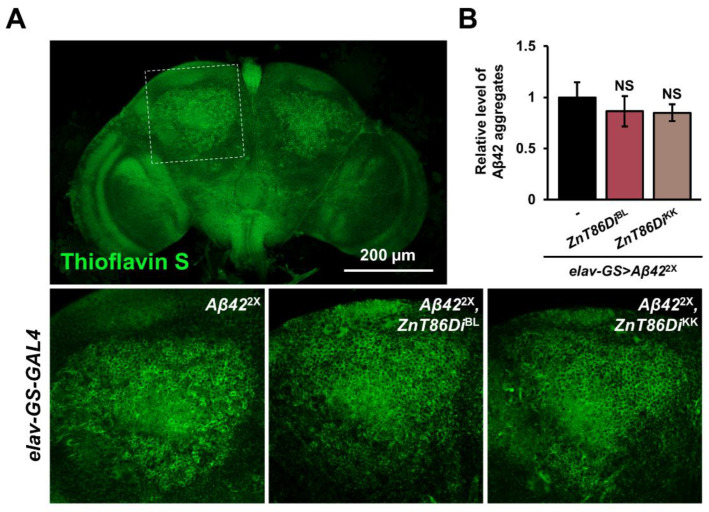
Silencing *ZnT86D* did not affect the deposition of amyloid beta (Aβ) plaques. (**A**) Representative confocal images showing Aβ plaques stained with thioflavin S of 30-day-old flies with overexpression of *Aβ42* and reduced expression of *ZnT86D* in neurons (*elav-GS > Aβ42*^2X^, *ZnT86Di*^BL^ and *elav-GS > Aβ42*^2X^, *ZnT86Di*^KK^) and age-matched control flies (*elav-GS > Aβ42*^2X^). (**B**) Relative level of Aβ42 aggregates of control group and experimental groups (Student’s *t*-test, *n* = 10, NS, not significant). Data are expressed as the mean ± SEM.

Deposition of excess Aβ aggregates is a major hallmark of AD [49], and it is known that zinc binds to Aβ peptides and promotes their aggregation in vitro [12,13]. In addition, Lang and colleagues demonstrated that silencing *dZIP1*, an ortholog of ZIP1 located in the cell membrane that transports zinc to the cytoplasm, in the neurons of a *Drosophila* AD model reduced the accumulation of Aβ deposits, and the authors attributed the results to the decreased zinc level in the brain by the downregulation [31]. Based on this, we hypothesized that silencing *ZnT86D* would affect the level of Aβ deposits in the brain of the *Drosophila* AD model. Therefore, we conducted thioflavin S staining and measured the amount of Aβ deposits after downregulating *ZnT86D* for 30 days in the neurons of adult flies. However, there was no significant difference between *ZnT86D* RNAi flies and control flies, indicating that knockdown of *ZnT86D* did not affect the accumulation of Aβ plaques (Figure 5A,B).

### 2.6. Silencing *ZnT86D* Did Not Affect the Susceptibility of Aβ42-Overexpressing Flies to Oxidative Stress

**Figure 6:**
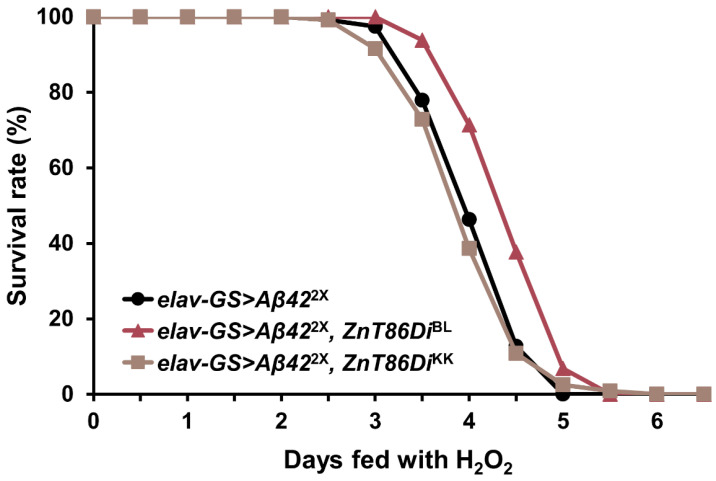
Silencing *ZnT86D* did not affect the susceptibility of *Aβ42*-overexpressing flies to oxidative stress. (A) Survival curve of neuron-specific *ZnT86D* RNAi (*elav-GS > Aβ42*^2X^, *ZnT86Di*^BL^ and *elav-GS > Aβ42*^2X^, *ZnT86Di*^KK^) and control (*elav-GS > Aβ42*^2X^) flies fed with hydrogen peroxide (H_2_O_2_) from an age of 20 days (Kaplan–Meier estimator and log-rank test, *n* ≥ 213).

Zinc is known to induce oxidative stress in the brain, and oxidative damage caused by an altered redox balance is a key pathophysiological feature of AD [50,51,52,53,54,55]. Therefore, we speculated that changes in the distribution of zinc in neurons by downregulating *ZnT86D* would affect the susceptibility of the *Drosophila* AD model to hydrogen peroxide (H_2_O_2_), which may mediate the enhanced Aβ42 cytotoxicity observed in *ZnT86D* RNAi flies. As shown in Figure 6 and Table 1, when 20-day-old flies were exposed to H_2_O_2_, the survival rate of *ZnT86Di*^BL^ flies was higher than that of control flies, while that of *ZnT86Di*^KK^ flies was not. These data suggest that the enhanced cytotoxicity of Aβ42 observed in *ZnT86D* RNAi flies was not the result of increased sensitivity to H_2_O_2_.

## 3. Discussion

ZnT7 is located mainly in the Golgi apparatus where it transports zinc from the cytoplasm into the Golgi apparatus [23]. Its physiological role has been actively studied using various models since its discovery in 2003. Interestingly, *ZnT7*-KO mice manifest metabolic abnormalities, including reduced body zinc status, body fat accumulation, glucose intolerance, and insulin resistance [25,26,41]. This suggests that *ZnT7* plays a crucial role in development, and in-depth studies on the role of *ZnT7* in this process are needed. Therefore, we silenced *ZnT86D*, a *Drosophila* ortholog of *ZnT7*, in a specific tissue by using the GAL4/UAS system, a powerful genetic tool in the field of research using *Drosophila melanogaster* as a model organism.

Surprisingly, neuron-specific *ZnT86D* knockdown had fatal effects on development, and RNAi flies showed extremely toxic phenotypes. In particular, the proportion of individuals eclosed to adults from the embryo, and the survival rate after eclosion decreased dramatically (Figure 1A and Figure 2B). These results are noteworthy in that adult flies did not eclosed from larvae when *ZnT86D* was overexpressed in neurons, which was attributed to localized zinc toxicity by the manipulation [30]. Therefore, the data suggest that the zinc distribution between the Golgi apparatus and cytoplasm is pivotal for normal neuronal development. Consistent with these results, the mushroom body, a center for learning and memory in *Drosophila*, was not formed normally in *ZnT86D* RNAi flies (Figure 2C). Indeed, zinc has been known to be strongly implicated in neurogenesis. For example, maternal zinc deficiency in mice impaired the expression of nestin, a marker of neural stem cells, in the offspring [7]. However, zinc intake during pregnancy boosted neuronal proliferation in the developing fetus [56]. Likewise, zinc homeostasis is crucial for normal neuronal development. 

In addition, the locomotor activity and lifespan of *ZnT86D* RNAi flies were dramatic ally decreased, consistent with the fact that neurodevelopmental disorders often accompany impaired motor function and increase the risk of premature death [36,37]. The RNAi flies also displayed symptoms similar to those of tremors, defined as rhythmic and involuntary movements of any body part [35]. According to a case report, 14-month-old male infants with infantile tremor syndrome showed zinc deficiency, suggesting an association between tremor and zinc [57]. Therefore, further studies are required to determine whether *ZnT86D* RNAi flies are zinc-deficient, thereby manifesting abnormal neurodevelopment and motility.

As shown in Figure 3A,B, we conducted AO staining to investigate whether the various defects caused by silencing *ZnT86D* were associated with cell death, and it was confirmed that cell death increased in the brains of *ZnT86D* RNAi flies. According to a recent study, *ZnT7* knockdown resulted in ER stress, and subsequently, JNK was activated in a *Drosophila* model of the malignant tumor *Raf*^GOF^*scrib*^−/−^ [58]. It is well known that JNK can induce apoptosis by upregulating pro-apoptotic genes [59]. Based on the results of this study we examined whether the increased cell death was due to ER stress through xbp1-EGFP, a marker of UPR, and anti-polyubiquitin immunostaining, but there were no significant changes in the *ZnT86D*-downregulated groups compared to those in the control group. This suggests that the increased cell death was not caused by ER stress, and the discrepancy between the results of Wei et al. (2021) and this study might be attributed to the difference in the model flies and investigated tissues. 

In 2012, Lang et al. (2012) demonstrated that genetic inhibition of *dZIP1* in neurons of a *Drosophila* AD model ameliorated Aβ pathology. When *dZIP1* was downregulated, brain zinc levels and the accumulation of Aβ42 fibril deposits were reduced [31]. Because zinc binds to the Aβ peptide and promotes its aggregation [12,13], Lang et al. (2012) concluded that the decrease in Aβ deposits was due to the reduced zinc concentration in the brain by *dZIP1* knockdown. In other words, silencing *dZIP1* in the neurons of the *Drosophila* AD model decreased the zinc level in the brain, leading to less oligomer formation, which in turn resulted in a decrease in the amount of Aβ plaques. By contrast, downregulation of *ZnT86D* in the neurons had no effect on Aβ accumulation (Figure 5A,B), suggesting that silencing *ZnT86D* does not elevate the level of cytosolic zinc enough to enhance Aβ aggregation.

As the increase in apoptosis and exacerbation of neurodegeneration by *ZnT86D* downregulation were not associated with the accumulation of Aβ42 plaques, we hypothesized that the lethal effects would have been manifested by increased cellular stress. In particular, since zinc is known to be associated with oxidative stress in the brain [49,51,53,54] and ZnT7 is known to protect mouse osteoblast MC3T3-E1 cells from oxidative stress-induced apoptosis through the PI3K/Akt and MAPK/ERK pathways [60], we postulated that the manipulation made neurons susceptible to oxidative stress. However, there was no significant change in the survival rate after exposure to H_2_O_2_ between *ZnT86D* RNAi flies and control flies, suggesting that the increase in cell death and exacerbation of neurodegeneration in the fly model brain were not associated with oxidative stress.

Then, what is the cause of the various toxic phenotypes manifested by *ZnT86D* downregulation in neurons? One possibility is that the increased cell death caused by *ZnT86D* knockdown in the developing neurons or in the neurons of the *Drosophila* AD model could have been associated with changes in metabolism. Indeed, *ZnT7*-KO mice show diet-induced glucose intolerance and insulin resistance [26,41], and it is known that *ZnT7* deficiency inhibits insulin-dependent Akt activation and glucose uptake [42]. Furthermore, *ZnT7* is expressed in the islets of Langerhans in the mouse pancreas, and it has been demonstrated that *ZnT7* overexpression in RIN5mf cells (rat insulinoma cells) increased insulin mRNA expression, insulin protein synthesis, and insulin secretion [61]. Insulin has anti-apoptotic activity [62], and insulin resistance is known to induce apoptosis [63]. Moreover, it is well known that the glucose is able to affect neurogenesis and hyperglycemia increases the risk of AD [64]. The insulin signaling pathway is conserved in *Drosophila*: IPCs located in the brain play the role of the pancreas, and DILP secreted from the IPCs play the role of insulin [65]. However, we found that the blood glucose level of *ZnT86D* RNAi larvae was not significantly different from that of the control group. This implies that brain cell death in our *Drosophila* AD model was not due to hyperglycemia. Furthermore, it raises the possibility that the function of *ZnT86D* in *Drosophila* metabolism is not completely identical to that of its mammalian orthologue. Future studies of *ZnT86D* function are expected to broaden our understanding of the role of zinc in animal cell metabolism as well as AD.

Another possible scenario is that perturbation of intracellular zinc distribution induced by *ZnT86D* knockdown affects cellular signaling, and consequently, adversely affects neuronal survival. A recent study on the importance of zinc fluctuations in cellular signaling strongly supports this possibility [66]. This study shows that at the single cell level, changes in intracellular zinc levels occur in response to subtle extracellular perturbations, and these changes directly correlate with changes in ERK and Akt activity [66]. Since ZnT7 is important for the movement of zinc from the cytoplasm to the Golgi [23,24], the cellular distribution of zinc would be changed by *ZnT86D* knockdown, which may affect the activity of the ERK and Akt signaling pathways. In general, ERK pathway is associated with cell growth, differentiation, and survival [67]. However, chronic ERK activation in pathological conditions is crucial to the pathology of AD and contributes to death signaling [68,69]. Meanwhile, Akt is a PI3K downstream factor that is involved in survival of various cells including neurons [70]. As chronic activation of ERK observed in neurons of AD patients [71], or decreased Akt activity are closely related to neuronal death [72,73,74], the decrease in survival of *ZnT86D* knockdown neurons in the development process or in stressful situations such as the presence of Aβ could be related to changes in ERK or Akt signaling pathways. The study about the alteration of signaling pathways in *ZnT86D* RNAi *Drosophila* is expected to provide important clues elucidating the neuroprotective role of *ZnT86D*.

Meanwhile, proteins that directly interact with ZnT86D may have been disrupted by *ZnT86D* downregulation. For example, ZnT7 activates alkaline phosphatases, zinc-requiring enzymes that are glycosylphosphatidylinositol-anchored to the cytoplasmic membrane, along with ZnT5 [75]. In addition, ZnT7 binds to CD40 and influences CD154-triggered p38 MAPK activity in B-lymphocytes [76]. Therefore, investigating the proteins that interact with ZnT7 will provide insights into this issue.

In conclusion, we demonstrated that *ZnT86D* plays a pivotal role in neuronal and wing development and in the pathogenesis of AD in *Drosophila*. When *ZnT86D* was downregulated in neurons, the embryo-to-adult survival rate, locomotor activity, and lifespan were dramatically reduced. These toxic phenotypes were accompanied by abnormal neurogenesis and neuronal cell death, which were not associated with ER stress and increased blood glucose level. In addition, knockdown of *ZnT86D* in neurons of a *Drosophila* AD model increased apoptosis and exacerbated neurodegeneration, but there was no significant change in the degree of Aβ plaque deposition and susceptibility to oxidative stress. Further studies need to be conducted on how the manipulation caused such fatal effects, and this endeavor would provide insights into the design of treatments for neurodevelopmental disorders and AD.

## 4. Materials and Methods

### 4.1. Drosophila Strains

Embryonic lethal abnormal vision (*elav*)-*GAL4* (BL458), patched (*ptc*)-*GAL4* (BL2017), engrailed (*en*)-*GAL4* (BL30564), glass multimer receptor (*GMR*)-*GAL4* (BL9146), *elav-Gene Switch-GAL4* (*elav-GS-GAL4*) (BL43642), and *UAS-ZnT86D* RNAi (*ZnT86Di*^BL^, BL44586) were obtained from the Bloomington *Drosophila* Stock Center. *UAS-ZnT86D* RNAi (*ZnT86Di*^KK^, v107388) was acquired from the Vienna *Drosophila* Stock Center. *MS1096-GAL4* and *UAS-Aβ42*^2X^ were gifts from Dr. M. Freeman (MRC Laboratory of Molecular Biology, UK) and Dr. Pedro Fernandez-Funez (University of Florida, USA), respectively.

### 4.2. Analysis of Survivability

Embryos from each genotype were collected on grape juice agar plates. Fifty age-matched embryos from each group were transferred to a vial containing a standard cornmeal medium and raised at 25 °C. The number of eclosed flies was counted, and the experiment was repeated three times. To measure the survival rate of adult flies, 20 flies of each genotype were kept in a vial at 25 °C. The flies were transferred to fresh media every 3 days, and the number of living flies was counted every 12 h.

### 4.3. Climbing Assay

The climbing assay was conducted as previously described [77] with minor modifications. Ten flies were collected in a vial prepared for the climbing assay and incubated for 1 h at 25 °C for environmental acclimation. After tapping the vial to move the flies to the bottom, individuals climbing to the midpoint of the vial within 10 s were counted. Ten trials were conducted for each vial and repeated four times for each genotype. Therefore, 40 flies were analyzed for each independently derived transgenic line. Climbing scores (the ratio of flies that reached the midpoint of the vial among all flies) were obtained, and the average scores of each group were compared.

### 4.4. Immunohistochemistry

Wing imaginal discs were dissected in phosphate-buffered saline (PBS) and fixed in 4% paraformaldehyde for 10 min. After washing with PBS containing 0.1% TritonX-100, the tissues were incubated with PBS containing 0.1% TritonX-100, 2% bovine serum albumin (BSA), and 2% normal goat serum (NGS) for 1 h at 25 °C. The samples were then incubated with mouse anti-engrailed antibody (1:200; 4D9; Developmental Studies Hybridoma Bank [DSHB], Iowa City, IA, USA) or mouse anti-wingless antibody (1:200; 4D4; DSHB) for 2 h at 25 °C. After washing with PBS containing 0.1% TritonX-100, the cells were incubated with Alexa-Fluor-488-labeled anti-mouse immunoglobulin G (IgG) secondary antibody (1:200; A-11001; Invitrogen, Carlsbad, CA, USA) for 1 h at 25 °C. After washing, the tissues were mounted using Vectashield mounting medium (H-1000; Vector Laboratories, Burlingame, CA, USA). 

For immunohistochemistry analysis of adult brains, adult flies were fixed in 4% paraformaldehyde containing 0.5% TritonX-100 (PBST) for 3 h. After washing with PBST, brains were dissected in PBST. The samples were incubated with PBST containing 2% BSA and 5% NGS for 3 h at 25 °C. The brains were then incubated with mouse anti-fasciclin-II antibody (1:200; 1D4; DSHB) or mouse anti-polyubiquitinated protein antibody [1:500; BML_PW0755-0025 (FK2); Enzo, Farmingdale, NY, USA] for 2 days at 4 °C. After washing, samples were incubated with Alexa-Fluor-488-labeled anti-mouse immunoglobulin G (IgG) secondary antibody (1:200; A-11001; Invitrogen) overnight at 4 °C. After washing with PBST, the brains were mounted using Vectashield mounting media. 

To detect cleaved caspase 1 (active DCP-1), the brains of adult flies were dissected in cold PBS and fixed with 4% paraformaldehyde for 30 min. After washing with PBS, samples were incubated with PBST containing 0.1% NGS for 3 h at 25 °C. The brains were then incubated with rabbit anti-cleaved *Drosophila* DCP-1 antibody [1:200; #9578; Cell Signaling Technology, Danvers, MA, USA] for 2 days at 4 °C. After washing with PBST, the samples were incubated with Alexa-Fluor-555-labeled anti-rabbit IgG secondary antibody (1:200; A-21429; Invitrogen) overnight at 4 °C. After washing, the brains were mounted using Vectashield mounting medium. All the samples were observed under a confocal microscope (LSM800; Carl Zeiss, Oberkochen, Germany).

### 4.5. Acridine Orange Staining

AO staining was performed as previously described [78]. The brains of third instar larvae were dissected in PBS and incubated with 1.6 × 10^−6^ M AO (318337; Sigma-Aldrich, St. Louis, MO, USA) for 5 min. The samples were rinsed twice with PBS and observed under a fluorescence microscope (Axiopot2; Carl Zeiss, Oberkochen, Germany).

### 4.6. Thioflavin S Staining

Thioflavin S staining was performed as previously described [79] with minor modifications. Adult flies were fixed in 4% paraformaldehyde containing 0.5% Triton X-100 for 3 h and washed with PBST. Brains were then dissected and incubated in 50% ethanol containing 0.125% thioflavin S (T1892; Sigma-Aldrich) overnight at 4 °C. After incubation in 50% ethanol for 10 min at 25 °C, the samples were washed with PBST and mounted using Vectashield mounting medium. Brains were observed under a confocal microscope (Carl Zeiss).

### 4.7. Histology

The flies were fixed in Carnoy’s fixative (ethanol:chloroform:glacial acetic acid at a ratio of 6:3:1) for 4 days at 4 °C and dehydrated with ethanol. The samples were washed with xylene three times for 30 min each and infiltrated with xylene:paraffin solution (1:1) for 1 h; the solution was changed every 20 min at 73 °C. After incubating for 2 h in paraffin changing the solution every 30 min at 73 °C, the brains were embedded and sectioned serially at 5 μm intervals to obtain frontal sections. The sections were stained with hematoxylin and eosin and examined under a light microscope (BX50; Olympus, Tokyo, Japan).

### 4.8. Oxidative Stress Test

The susceptibility of each group to oxidative stress was estimated using hydrogen peroxide (H_2_O_2_). The flies of each genotype were collected immediately after eclosion and fed RU486 for 20 days. They were then starved for 6 h and transferred to vials with a medium containing 1% H_2_O_2_ and 5% sucrose. The number of live flies was counted every 12 h.

### 4.9. Measurement of the Levels of Glucose and Protein in Larval Hemolymph

Quantification of glucose levels in the larval hemolymph was performed as described [80,81]. Seven male larvae of each group were washed twice with water and dried on tissue paper. The cuticles at the mouth part of the larvae were then carefully torn and placed in a perforated 0.5 mL tube within a 1.5 mL tube. The tube was centrifuged at 12,000 rpm for 10 s using a microcentrifuge to obtain hemolymph. The hemolymph was diluted 1:30 in PBS and heat treated at 70 °C for 5 min. Glucose level was measured using the Glucose Assay Kit (GAGO20; Sigma-Aldrich) and glucose concentration was calculated from a standard curve generated with glucose standards. The amounts of glucose were normalized to the protein level measured by detergent compatible (DC) protein assay. The DC Protein Assay Kit (500-0111; Bio-Rad, Hercules, CA, USA) was used to measure the protein level in hemolymph before heat treatment. Protein concentration was calculated from a standard curve generated with the dilutions of BSA standards.

### 4.10. Statistical Analysis

Except for the survivability data of adult flies and the susceptibility of a *Drosophila* AD model to oxidative stress, all data were analyzed with Student’s t-test or one-way ANOVA followed by Tukey–Kramer multiple comparison test for post-hoc analysis. GraphPad Prism version 8.0.1 (GraphPad Software, San Diego, CA, USA) was used to reveal statistical significance, and differences were considered significant at *p*-values less than 0.05. The Kaplan–Meier estimator and log-rank test were used to analyze the survival rate of adult flies and the susceptibility of a *Drosophila* AD model to oxidative stress using the Online Application for Survival Analysis of Lifespan Assays 2 ([82]; https://sbi.postech.ac.kr/oasis2/, accessed on 6 September 2022). Body size, AO-positive cells, the area of neuronal loss, and the levels of Aβ42 aggregates were quantified using Photoshop.

## Figures and Tables

**Table 1:** Silencing *ZnT86D* did not affect the susceptibility of *Aβ42*-overexpressing flies to oxidative stress.

			Log-Rank Test					
			*p*-Value			% Change		
Strain	No. of Flies	Mean Lifespan (h)	vs. A	vs. B	vs. C	vs. A	vs. B	vs. C
Trial 1								
*elav-GS > Aβ42*^2X^ [A]	117	100.00 ± 1.15	-	7.0 × 10^−8^	0.4081	-	−8.46	2.01
*elav-GS > Aβ42*^2X^, *ZnT86Di*^BL^ [B]	116	109.24 ± 1.14	7.0 × 10^−8^	-	2.2 × 10^−8^	9.24	-	11.44
*elav-GS > Aβ42*^2X^, *ZnT86Di*^KK^ [C]	118	98.03 ± 1.33	0.4081	2.2 × 10^−8^	-	−1.97	−10.26	-
Trial 2								
*elav-GS > Aβ42*^2X^ [A]	96	94.13 ± 0.96	-	<1.0 × 10^−4^	2.7×10^−3^	-	−10.51	3.83
*elav-GS > Aβ42*^2X^, *ZnT86Di*^BL^ [B]	115	105.18 ± 1.07	<1.0 × 10^−4^	-	<1.0 × 10^−4^	11.74	-	16.02
*elav-GS > Aβ42*^2X^, *ZnT86Di*^KK^ [C]	119	90.66 ± 0.76	2.7 × 10^−3^	<1.0 × 10^−4^	-	−3.69	−13.80	-

## Data Availability

The data presented in this study are available on request from the corresponding author.
